# Test-re-test reliability and dynamics of the Fukuda–Unterberger stepping test

**DOI:** 10.3389/fneur.2023.1128760

**Published:** 2023-03-29

**Authors:** Simone Hemm, Denise Baumann, Vasco Duarte da Costa, Alexander Andrea Tarnutzer

**Affiliations:** ^1^School of Life Sciences, Institute for Medical Engineering and Medical Informatics, University of Applied Sciences and Arts Northwestern Switzerland, Muttenz, Switzerland; ^2^Neurology, Cantonal Hospital of Baden, Baden, Switzerland; ^3^Faculty of Medicine, University of Zurich, Zurich, Switzerland

**Keywords:** spatial orientation and navigation, perceived straight-ahead, inertial measurement units, Fukuda stepping test, Unterberger test

## Abstract

**Background:**

The Fukuda-stepping-test (FST), i.e., repetitive walking on the spot while blindfolded, has been proposed as a means to assess the integrity of the vestibular pathways. While its sensitivity to detect abnormalities in patients is limited, it may be useful in studying the physiology of the subjective-straight-ahead (SSA). Considering reported systematic shifts in SSA in humans, we hypothesize that such asymmetries arise from individual differences in the orientation/configuration of the macular organs and in central processing of vestibular input. We hypothesize that such asymmetries are stable over time in individual subjects. Alternatively, such asymmetries may arise from random noise in the sensory/motor systems involved, demonstrating low reproducibility over time.

**Materials and methods:**

Twenty-four subjects walked on the spot over 60 s while blindfolded (*n* = 6 trials per subject). Using an inertial measurement unit (IMU) placed at the chest, angular deviations were recorded and compared to manually-measured final positions. Both static (direction, magnitude) and dynamic (time-to-onset of deviation, pattern of deviations) parameters were retrieved from the yaw slopes.

**Results:**

Significant deviations were found in 15/24 participants for the manual measurements (leftwards = 8; rightwards = 7), whereas when using the IMU-sensor 13/24 participants showed significant shifts (leftwards = 9; rightwards = 4). There was a high correlation (0.98) between manually measured rotation angles (average absolute deviations = 58.0 deg ± 48.6 deg; intra-individual variability = 39 deg ± 24 deg) and sensor-based yaw slopes (1.00 deg/s ± 0.88 deg/s; 0.67 deg/s ± 0.41 deg/s). Relevant yaw deviation was detected 22.1 s ± 12.3 s (range = 5.6 s-59.2 s) after the onset of marching (no relevant yaw-deviation in 15/139 measurements), showing a mostly linear behavior over time.

**Conclusion:**

We observed significant inter-individual variability in task performance in the FST, reproducing findings from previous studies. With test-re-test reliability being moderate only, but at the same time observing a preference in the side of shifts in most trials and subjects, we conclude that likely both individually varying estimates of straight-ahead and random noise contribute to the pattern of angular deviations observed. Using an IMU-sensory based approach, additional dynamic parameters could be retrieved, emphasizing the value of such a quantitative approach over manual measurements. Such an approach may provide useful additional information to distinguish patients from healthy controls.

## 1. Introduction

Spatial orientation and navigation require internal estimates of straight-ahead, self-motion and direction of gravity. Therefore, input from various sensory systems, including the vestibular organs (semicircular canals and otolith organs), hearing, vision and proprioception (skin pressure sensors, joint receptors) are centrally combined (1, 2) and weighted in an optimal fashion (3). The otolith organs, however, are the only sensors that directly measure linear acceleration including gravity, and the proper functioning of navigation critically depends on the integrity of the vestibular organs and of the central vestibular pathways. If these pathways or the vestibular organ on one side are damaged, patients will show systematic deficits of postural control and walking (4). Specifically, subjective straight ahead (SSA) is biased and they tend to deviate toward the affected ear when walking (5). To assess such lateralized vestibular deficits at the bedside, repetitive walking on the spot while blindfolded has been proposed by Unterberger in 1938 (6). Later on, Fukuda has slightly modified this stepping test (referred to as Fukuda stepping test (FST)) and has concluded that it is sensitive for detecting peripheral vestibular deficits and thus deviations in the SSA. He reported cut-off values of 30 deg (for 50 steps) and 45 deg (for 100 steps) of body rotation to allow the distinction between bilaterally normal vestibular function from lateralized peripheral-vestibular deficits (7). Its value in accurately and precisely detecting vestibular tone imbalance at the bedside, however, has been questioned, and studies have demonstrated limited sensitivity and specificity of the FST. While Fukuda reported ipsilesional deviations in single patients with middle ear disease, others have found inconsistent results in patients with various chronic peripheral-vestibular disorders, showing no correlation between the side of the lesion and deviations in the FST (8–13). These inconsistencies have been linked to emerging central compensation (11), and it has been emphasized that the vestibular tone imbalance assessed by the FST includes both asymmetries originating from lateralized peripheral vestibular lesions and central compensatory mechanisms, thus being under control of central adaptation (14). While it has been recommended not to use the FST as a screening test in patients with suspected vestibular disorders (8, 11), it may be of value in assessing the physiology of the SSA resulting from multisensory integration in healthy human subjects and thus reflecting vestibular tone (a)symmetry in the yaw-plane.

Noteworthy, also in healthy human subjects with normal vestibular function, deviations in the FST are well-known, with significant inter-individual spread (15) and exceeding cut-off values originally proposed by Fukuda (7). Previously, we have shown that the SSA is significantly biased by the preceding whole-body roll orientation (16). Specifically, after lying 5 min in either right-ear-down or left-ear-down orientation (with eyes closed), walking straight-ahead blindfolded demonstrated significant deviations toward the side of the resting position. This underlines the impact of central adaptational mechanisms on the SSA. Remarkably, participants demonstrated deviations from walking straight-ahead at baseline as well, with an overall-tendency toward the left side. Similarly healthy human subjects tend to walk in circles when blindfolded or in a territory without visual orientation cues, as reported by Souman et al. (17). Data presented by Souman suggest that within one period of measurement, deviations are consistently to one side (i.e., resulting in clockwise or counterclockwise circles). However, in this study no serial measurements were obtained, allowing no conclusions on the test-re-test reliability in individual subjects.

Considering the reported deviations in walking straight-ahead when blindfolded and the deviations in the FST in individual subjects, we hypothesize that subjects show systematic deviations when asked to keep aligned with the SSA when removing visual and auditive cues. We propose that such asymmetries arise from individual differences in the orientation and the configuration of the macular organs and in central processing of vestibular input. Thus, we therefore predict such asymmetries to be stable over time in individual subjects. This assumption is supported by previous observations by Reiss and Reiss (18). Alternatively, such asymmetries may arise from random noise in the sensory systems involved. In this case, however, a low reproducibility over time is predicted.

Traditionally three parameters are assessed after FST performance on a circular grid placed on the floor: quantifying the angle of rotation, the angle of displacement and the distance of displacement [see, e.g., (19)]. Such a setup, however, is possibly prone to measurement inaccuracies and also does not provide any information about the dynamics of changes in body orientation while performing the task. By quantitatively assessing body position in space (using inertial measurement unit (IMU) sensors), we will be able to assess both the dynamics during the FST and to obtain more precise measurements (especially regarding the angle of rotation) after finishing the task as well.

## 2. Materials and methods

### 2.1. Ethical approval

All subjects provided written informed consent after a full explanation of the experimental procedure. The study was approved by the Ethikkommission Nordwest-und Zentralschweiz (EKNZ, ID = 2020-01712) on research involving humans. The research project was conducted in accordance with university policies, the Federal Act on Data Protection, the Declaration of Helsinki (except for registration in a database), the principles of Good Clinical Practice, the Human Research Act (HRA) and the Human Research Ordinance (HRO).

### 2.2. Subjects

Twenty-five healthy, adult human subjects (9 females, 16 males, age [mean ± 1 standard deviation (SD)]: 29.4 ± 8.7 years, range = 20–60 years) were recruited for the study. All participants were screened for pre-defined exclusion criteria including a history of vestibular or gait/balance disorders, intake of centrally-active medication (as antidepressants or neuroleptics) or sensorimotor deficits. Subjects weighted between 50 kg and 121 kg with an average height of 164 cm and 179 cm for female and male subjects, respectively. Twenty-two out of the 25 subjects reported to be right-handed. One of those 25 subjects (#5) was excluded due to severe fatigue from previous exercises, so that the exercise could not be performed properly. Specifically, the participant had difficulties lifting his legs properly and keeping his balance.

### 2.3. Experimental setup

**Figure 1:**
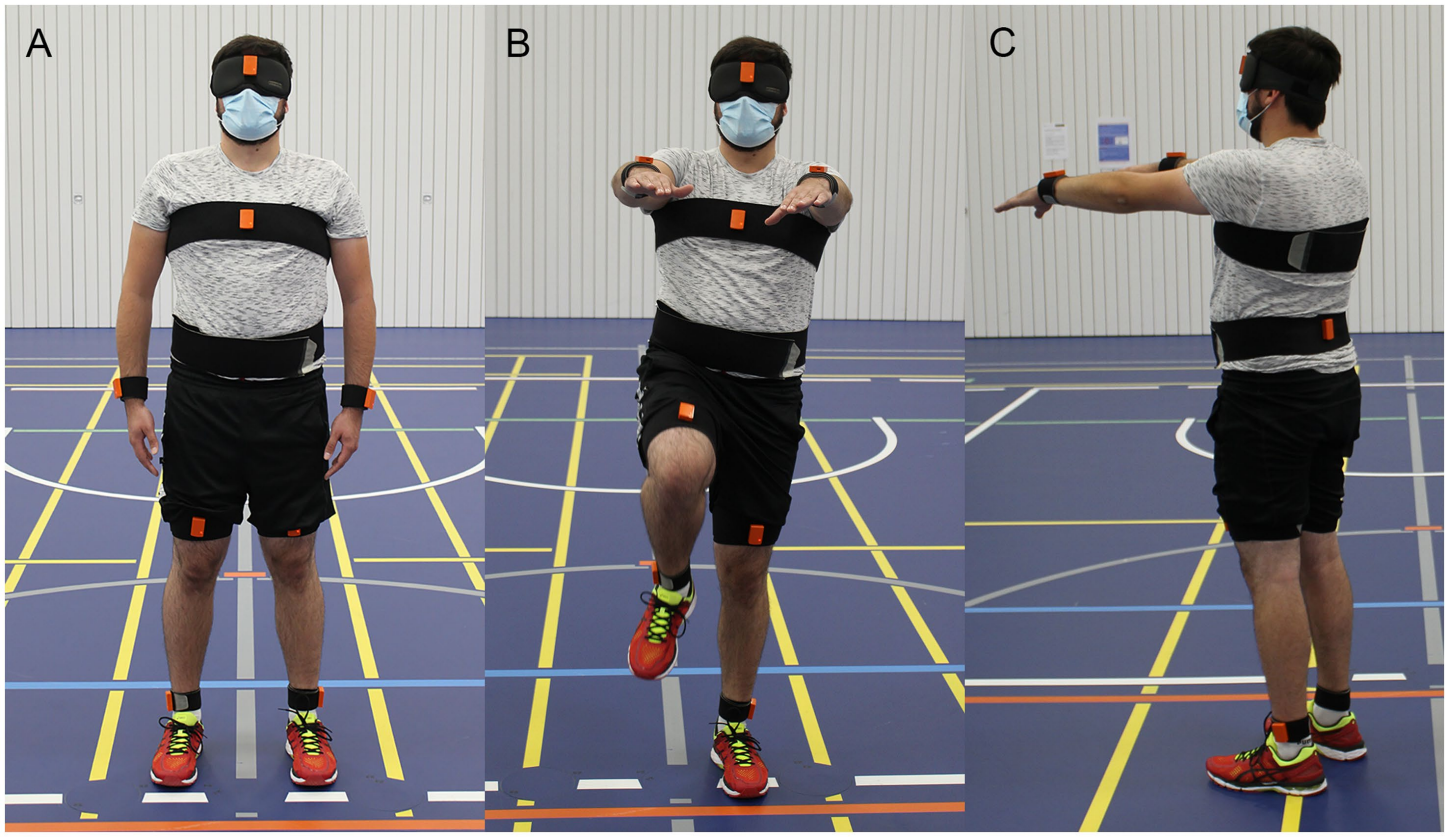
Illustration of the Fukuda/Unterberger stepping test (FST), here referred to as “marching test.” Subjects stood in neutral pose **(A)** and then started marching at the spot with the arms elevated for 60 s **(B)**. The final position and orientation of the subject at the end of the trial **(C)** was recorded. In this example the subject rotated to the right-hand side while being displaced anteriorly.

The trials were performed in a double sports hall. The origin of coordinates (called “starting point”) for the Fukuda/Unterberger stepping test, hereafter referred to as marching test, was one of the sports hall’s markings’ circles. All trials were performed while the subject was equipped as shown in our previous publication [see figure 1 from previous publication (16)]. The subjects wore a sleeping mask and earmuffs to eliminate visual cues during marching and to reduce auditory feedback for orientation in space, respectively.

### 2.4. Experimental paradigm

Each participant performed the marching test six consecutive times. The subjects were accompanied by the experimenter to allow interventions when needed. At the beginning of each repetition the subjects stood in neutral pose in the center of a circle and were aligned to a central line (Figure 1A). The neutral pose was defined as the subject standing upright with the feet parallel to the hips and the palm of the hands turned toward the body. Next, the subjects elevated their arms 90 deg around the shoulder to the front and started marching on the spot (Figure 1B). While marching, the legs had to be lifted until the thigh reached approximately a 90 deg angle relative to the trunk. The subjects were asked to march for 1 min with a cadence of 60–80 steps per minute. The end of marching was indicated by a shoulder tap or a loud signal, whereafter the subject stopped marching and remained at the final position (Figure 1C). The final position and the angular orientation of the subject was marked on the floor. Between the repetitions the subject was allowed to rest and remove the blindfold, but no feedback about task performance was given. All parameters (heading rotation, displacement in x and y at end of trial) were measured manually with a goniometer and measuring tape at the end of the experiment.

### 2.5. Inertial measurement unit based motion tracking

Motion was measured with nine inertial motion trackers (MTw Awinda, Xsens Technologies B.V., Enschede, Netherlands). For later determination of eventual irregularities, the feet and floor were also filmed using a GoPro Hero 6 camera (GoPro Inc., San Mateo CA, United States) during all trials. The Awinda motion tracker is composed of a three-dimensional (3D) accelerometer, 3D gyroscope, 3D magnetometer, barometer and a thermometer. The coordinate system of the estimated sensor orientation can be reset so that all sensors are aligned to the same orientation (20). Inertial motion trackers were placed at both ankles, both thighs, lower back, chest, both wrists and at the forehead with Velcro® patches and bands, resulting in a total of nine sensors attached. The individual sensors were placed at the same defined body parts for every participant. The forehead sensor was directly placed onto the sleeping mask at the height of the nasion. The chest sensor was either positioned on a chest mount (Chesty, GoPro Inc., San Mateo CA, United States) or on a Velcro^®^ band, which was wrapped around the subject’s chest. Signals were recorded at a sampling rate of 100 Hz. Before each individual measurement, the sensor orientation of all motion trackers was reset while the subject stood in neutral pose.

#### 2.5.1. Data analysis

The sensor output data used for further processing do not represent direct instantaneous inertial measurements, but reflect precomputed data outputted by a specific Kalman filter by the IMU manufacturer (XKF3-hm, Xsens Technologies B.V., Enschede, Netherlands). For a detailed analysis of the performance of MTw Anwinda sensors and the built-in algorithms used see Paulich et al. (figure 6 in their publication) (20). Magnetometer data were only used for the XSens data calibration based on a specific Kalman filter [see (20)], but not for the calculation of angular deviation. A preliminary data analysis based on the angular rotation detection provided by the XSens, which includes the magnetometer data, demonstrated not satisfying results. Specifically, the starting angle was not always zero and the orientations could be mirrored depending on the starting orientation to the earth magnetic field. This contradicts the precision described in the white paper by Paulich et al. of 1.5°, where results from different measurement systems were compared (20), but no comparison toward manual measured values was made. Orientation taking into account magnetometer data is sensitive toward magnetic field distortions. This could potentially explain the observed lower precision and mirror effects. Thus, discarding magnetometer data for our data analysis, no calculating of angular rotation based on the IMU data (i.e., IMU displacement) was possible. Instead, IMU angular velocity was calculated based on a linear regression of the chest IMU sensor (see section “processing of sensor data” for details).

Calculations (fusing accelerometer and gyroscope data, see further below) were performed and results plotted using MATLAB (version R2021a; The MathWorks Inc., Natick, MA, United States). A preliminary analysis of the data from the different IMU trackers demonstrated that the chest motion tracker showed the steadiest behavior in terms of body orientation during walking. Thus, for further analyses we used data from this sensor only. The statistical analyses were performed with RStudio Version 1.4 (RStudio, Boston, MA, United States) and R Version 4.03 (R Core Team, Vienna, Austria). Statistical comparisons were conducted with the R library rstatix (21). Intraclass correlation was investigated using Excel 2021 (Microsoft, Redmond, WA, United States).

#### 2.5.2. Processing of sensor data

**Figure 2:**
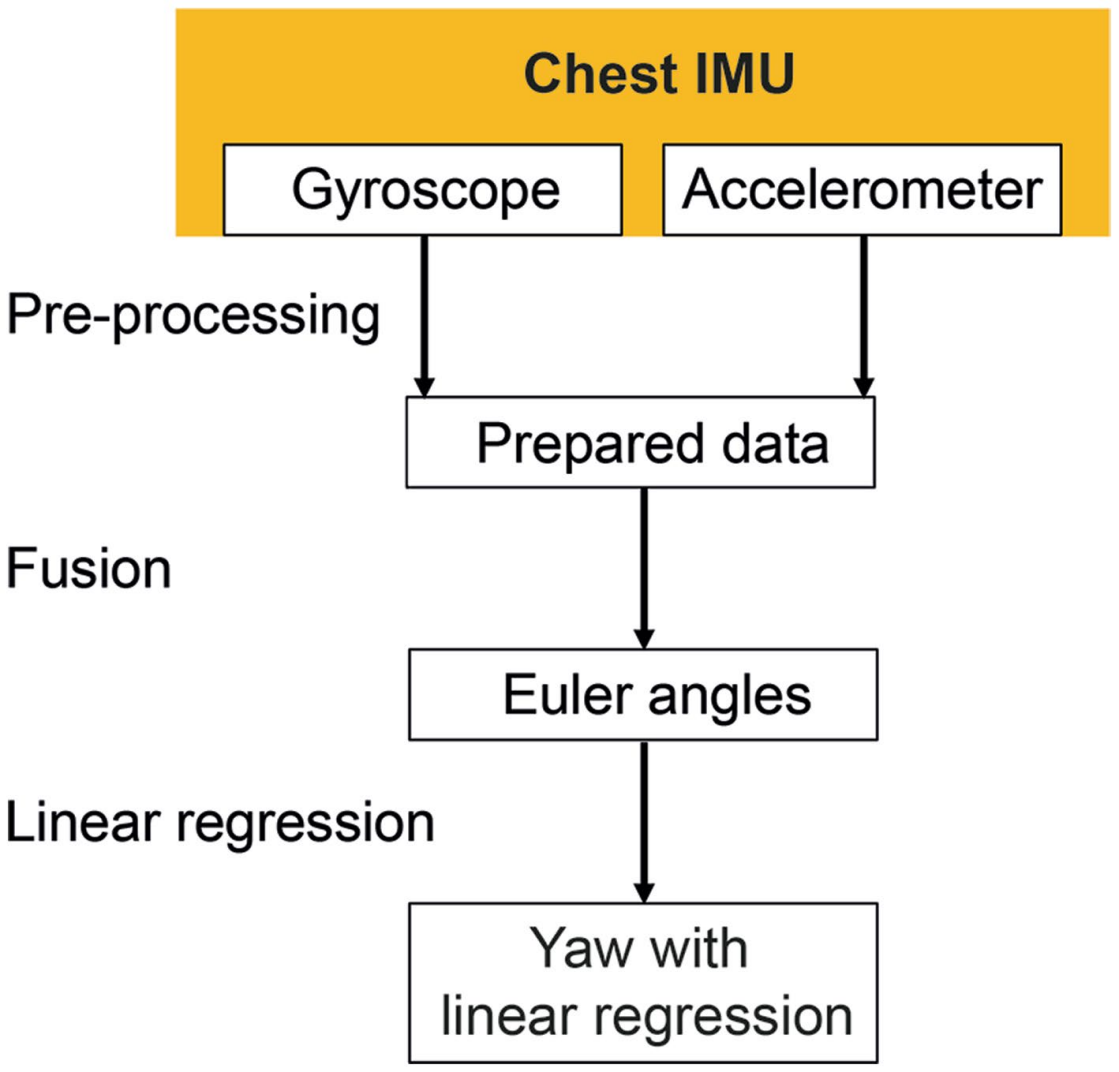
Signal processing of the chest inertial measurement unit (IMU). Missing data were interpolated during the pre-processing phase. Gyroscope and accelerometer data were fused to calculate Euler angles in three-dimensional space. The linear regressions of the resulting yaw signal were calculated to identify the resulting rotation angle.

A schematic overview of the signal processing of the sensor data is shown in Figure 2. Missing data from the measurements were interpolated using modified Akima Cubic Hermite interpolation and the signals were scaled to standardized measurement and time units. The experiment’s start and end were defined by determining the first and last step of a walking sequence. To find every step in the ankles’ vertical acceleration signals, a peak threshold algorithm with a minimum peak distance of 0.4 s and a minimum peak prominence of 4 m/s^2^ was used. A step was considered the first step of the walking sequence when at least five consecutive acceleration peaks were recognized within a defined time frame. The first step of every walking segment was identified by comparing the events of both legs. The last step was defined with the same conditions but in reverse direction. Data more than 2 s before the first step and more than 2 s after the last step were removed.

The heading direction or yaw was calculated to describe rotations around the cranio-caudal axis of the subjects. The orientation of the chest sensor was estimated by fusing acceleration (accelerometer) and angular velocity (gyroscope) data using a Kalman filter [MATLAB function imufilter *imufilter (‘SampleRate’,100,‘DecimalFactor’,1)*]. No magnetometer data were considered as mentioned above. The calculated orientation was transformed to Euler angles relating to the medio-lateral (pitch), anterior–posterior (roll) and the cranio-caudal (yaw) body axes. Linear regression of orientation of the chest around the cranio-caudal axis (yaw) was computed. The inclination of the determined regression line is further being referred to as “yaw slope.”

**Figure 3:**
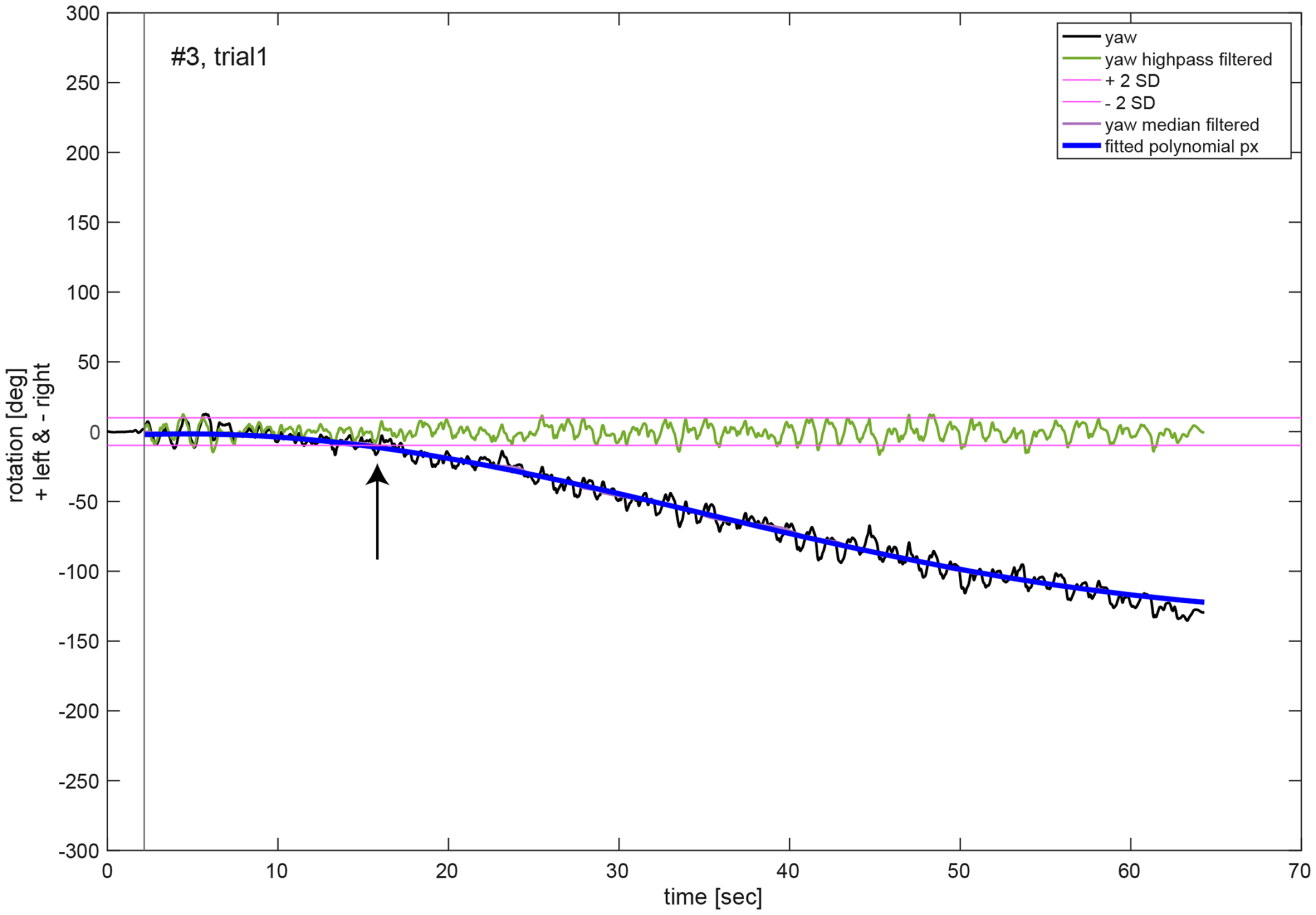
Illustrative example of the movement dynamics analysis. The oscillations in the chest heading orientation (yaw, black line) result from upper body twists while marching. The initial measurement phase (start of data recording to onset of marching as indicated by a black vertical line) was excluded for analysis of movement dynamics. A polynomial function *p_x_* (blue line) of 5th order of Equation (1) was fit to the median filtered yaw signal (violet line). The black arrow denotes the time of relevant change in heading orientation determined as median filtered yaw exceeding two SD of the high pass filtered (green line, Butterworth of sixth order, cut off frequency 1 Hz) yaw signal.

For the analysis of the movement dynamics, the initial measurement phase without any notable rotation around the cranio-caudal body axis (yaw rotation) was excluded. The starting point of the measurement (black vertical line, Figure 3) was set to the first point in time when the absolute yaw value was larger than two degrees. To remove oscillations due to upper body twists during marching movement, yaw rotation was filtered (median filter, window size 1,500, MATLAB function medfilt1). A polynomial function *p_x_* of 5th order (Equation S1, see Supplementary material) was fitted to the filtered yaw data for each trial (MATLAB function polyfit; blue line, Figure 3). Quantitative analysis of the chest heading orientation development over measurement time was described by this polynomial function. Additionally, the median filtered yaw was used to determine the time of relevant deviation of yaw rotation from the initial marching direction of the participant, whereas its onset was defined as the time point when the median filtered yaw rotation was larger than two standard deviations (SD) of the rotation (threshold indicated as purple line in Figure 3) due to upper body twists which were determined in the high pass filtered yaw signal (green line, Figure 3). High pass filtering was performed to exclude overall chest heading rotation deviation. It was achieved by a sixth order Butterworth filter, cut off frequency 0.1 Hz (MATLAB functions butter and filter).

For the analysis of the yaw deviation dynamic behavior per participant, the average function f_av_ and the 95% confidence intervals (CI) of all polynomial functions *p_x_* for all of 6 measurements were computed in Matlab (see Supplementary material for Equation (S2)). A polynomial of 5th order of Equation (S1) *p_av_* was fitted to the computed average function f_av_. Finally, for analysis of yaw deviation dynamic behavior over all participants, the average function f_av_av_ and 95% confidence intervals of all average functions f_av_ per participant were computed in Matlab similar to (S2). A polynomial of 5th order of Equation (S1) *p_av_av_* was fitted to the computed average function f_av_av_.

Trials with a yaw slope exceeding the three times interquartile range for yaw slope distributions of all trials were considered outliers and were removed.

### 2.6. Statistical analysis

Descriptive statistics were used to identify those participants with significant angular rotations at the end of the trials, focusing on the calculated individual average angular deviation and the resulting 95% CI. Individual participants were considered to deviate significantly in case the 95% CI of measured angular rotations did not include zero angular deviation. The yaw slope as a measure to describe upper body rotations was compared to the manually measured angles in a correlation analysis, providing a Pearson correlation coefficient (see Supplementary material for detailed description). As a measure for the test–retest reliability, we calculated both the intra-individual trial-to-trial variability (i.e., the precision, expressed as 1 SD over individual trials) and the intra-class correlation coefficient based on a single-rating (*k* = 1), absolute agreement, two-way mixed effect model [ICC (A,6) following the nomenclature introduced by McGraw and Wong (22)].

## 3. Results

### 3.1. Manual measurements – Individual angular deviations

**Figure 4:**
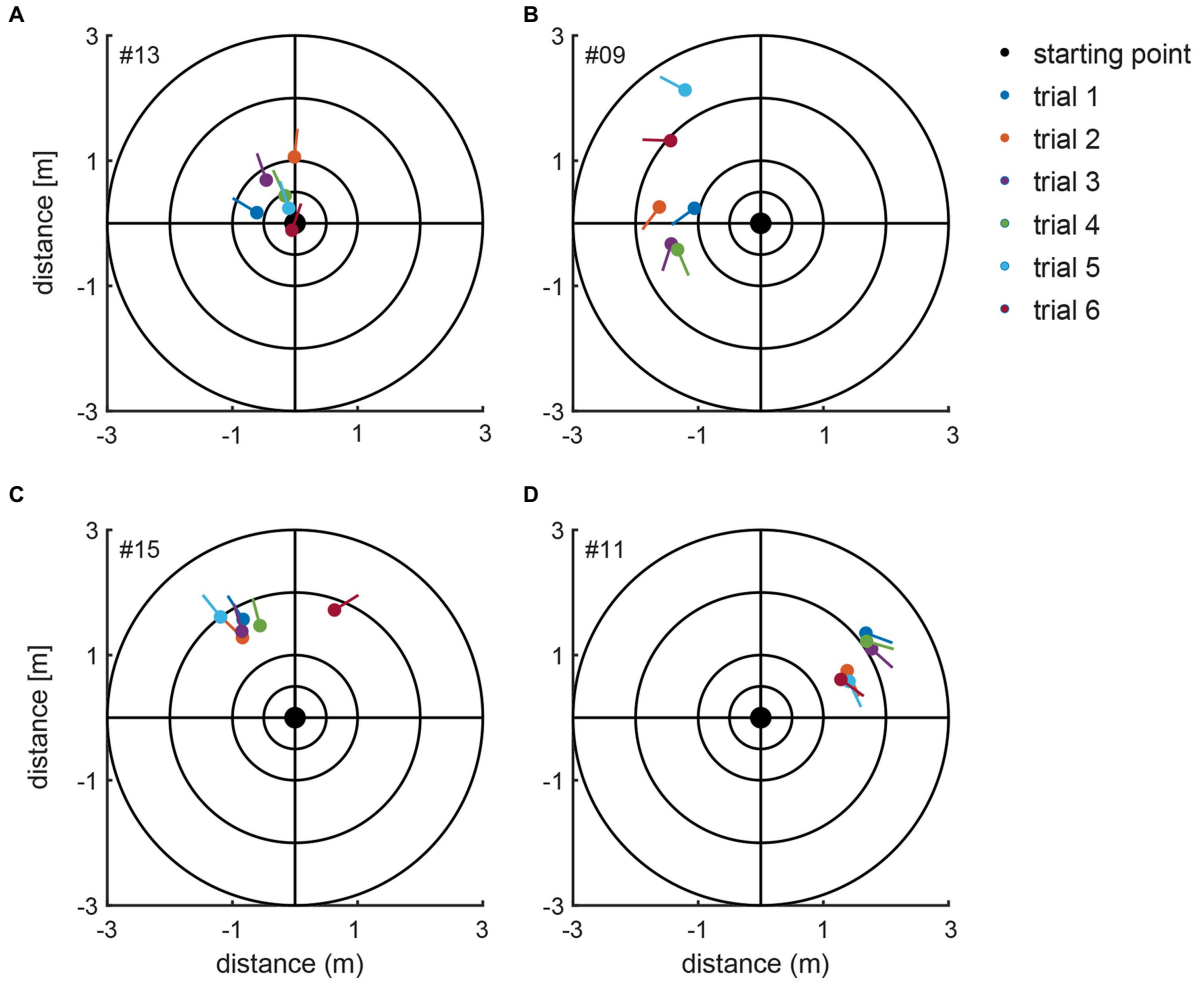
Illustration of the manually measured angular and linear deviations at the end of the marching test (as seen from above). Individual trials from four representative subjects (#13, #09, #15, and #11) are shown in panels **A–D**. The dots present the end position, and the lines indicate the final orientation. The initial subject orientation was in the center oriented toward the positive y-directions. The inlet indicates the color code for the individual trials.

For the manually measured end positions subjects deviated by 58.0 deg ± 48.6 deg on average (between 0 and 282 deg) when considering absolute values, i.e., not taking into account the direction of deviation (left vs. right). When the direction of deviation is considered (positive values indicate a deviation to the left, negative values to the right), an average deviation for all subjects of 4.1 deg ± 75.7 deg with a range of 485 deg (−282 deg to +203 deg) was found. Thus, we noted significant inter-individual variability for both the direction and the magnitude of angular deviations. Manually measured end positions after individual trials are shown in Figure 4 for four representative subjects, demonstrating distinct marching patterns. Whereas subject #13 (Figure 4A) turned to the left-hand side with an average of 16 deg ± 27 deg, subject #9 (Figure 4B) walked more to the left side and the resulting angular deviations were more variable (130 deg ± 50 deg, average ± 1SD). In the third subject (#15) (Figure 4C) measured angular deviations were less spread and this subject turned leftwards with small scatter (mean 29.8 deg ± 13 deg, trial 6 was determined to be an outlier and thus was excluded). Subject #11 (Figure 4D) deviated to the right side and was displaced slightly anterior, with little variability on resulting angular deviations (−130 deg ± 21 deg).

Considering all FST trials with a deviation of more than 10 deg as deviating significantly [as previously proposed by (18)], 126/139 trials (with five trials being considered outliers and thus removed) demonstrated significant deviations (63 trials (45.3%) each deviating leftward and rightward, respectively). The remaining 13/139 trials (9.4%) showed minor or no deviations.

### 3.2. Manually measured angle inter- and intra-individual variability

**Figure 5:**
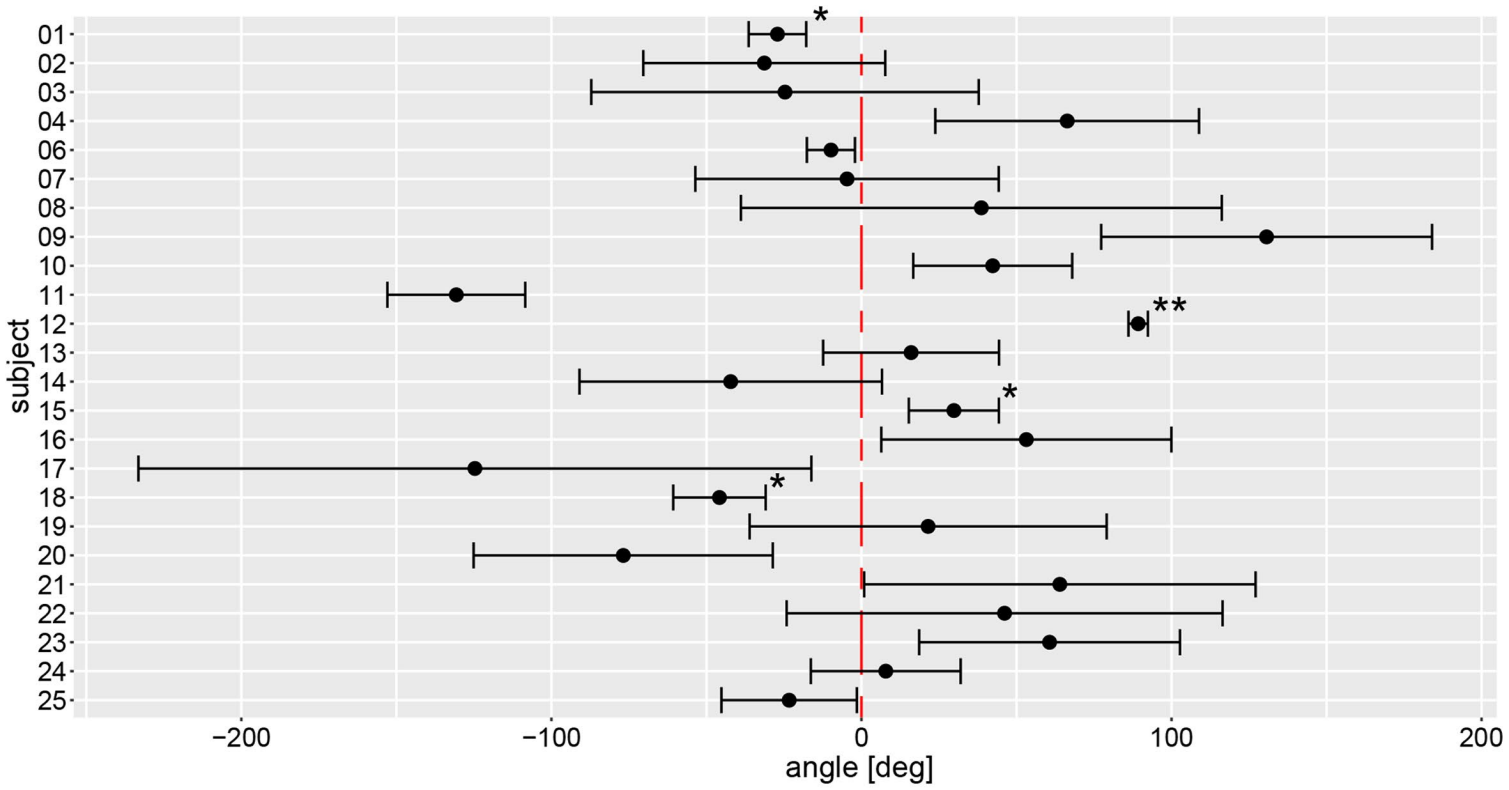
Illustration of individual average angular deviations (including 95% confidence levels) of manually measured angles for a total of 6 trials. The average values and 95% confidence levels were calculated after outliers exceeding a three times interquartile range for yaw slope distributions were removed (Figure 7). Individual average traces were marked with a star (“*”) to indicate that an outlier was removed, the number of stars reflects the number of outliers removed. The distribution of a participant is not significantly different to zero when the whisker crosses with the red, dashed line indicating no angular deviation. A negative angle corresponds to a rotation to the right, a positive to the left.

Based on the manual measurements, average individual angular deviations deviated significantly from zero degree in 15 out of 24 subjects, as shown in Figure 5. Eight participants significantly deviated to the left side, seven to the right side. Only subject #7 had an equivalent deviation to the left and the right side with a mean value close to zero. All other subjects showed at least a tendency to deviate toward one direction. The largest angular variability between the different trials was seen in subject #17, a very low inter-trial variability was noted in subjects #1, #6, and #12. Noteworthy, for subjects #1 and #12 outliers have been removed, potentially contributing to lower trial-to-trial variability.

On a trial-by-trial basis, we found that 9/24 subjects deviated to the same side on all 6 trials, whereas all other subjects deviated to the other side on 1 (*n* = 8), 2 (*n* = 6) or 3 (*n* = 1) trials. Intra-individual trial-to-trial variability (with outliers exceeding a three times interquartile range for yaw slope distributions being removed, see further below) varied considerably between participating subjects (range = 3.0–103.4 deg), with an average value of 38.9 deg ± 24.1 deg. The intra-class correlation coefficient for the six repetitions (no outliers removed) showed moderate reliability (0.61 (95% CI = 0.53–0.69, *p* < 0.001)) according to Koo et al. (23).

### 3.3. Sensor measurements – Individual angular deviations

**Figure 6:**
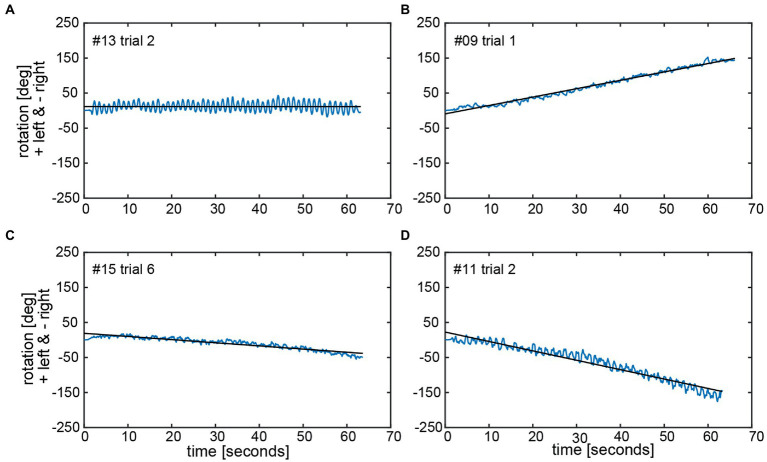
Chest heading orientation (‘yaw’, blue line) over time with a fitted linear regression (black line) are shown for the same four subjects (#13, #09, #15, and #11) as used in Figure 4 in panels **A–D**. The oscillations in the yaw result from upper body twists while marching. The slope of the linear regression (‘yaw slope’) was used in statistical investigations as an estimation of heading direction. Negative yaw corresponds to chest heading rotation to the right, positive to the left.

**Figure 7:**
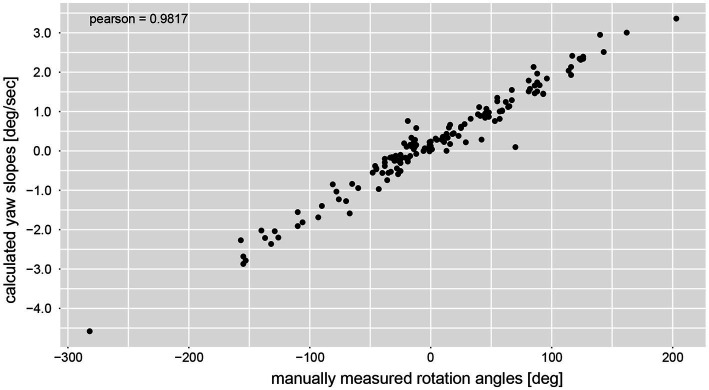
Correlation analysis between manually measured rotation angles (i.e., as angular displacement [deg]) and the yaw slope (slope of the linear regression of the chest heading orientation; i.e. as angular velocity [deg/s]).

The calculated chest heading orientations (‘yaw’) with fitted linear regressions are illustrated in Figure 6 for one trial for the same four subjects already considered in Figure 4. The example from subject #13 (Figure 4A) shows only a small deviation to the right-hand side (slope of linear regression = −2.436E-05). The resulting heading direction of subject #9 (Figure 4B) during the first attempt indicates an upper body rotation to the left-hand side. The subjects in Figure 4C (subject #15) and Figure 4D (subject #11) show heading directions to the right, with subject #11 demonstrating a larger angle of rotation than subject #15. A correlation analysis of all individual manually measured angles of rotation and all calculated sensor-based yaw slopes (inclination of the regression line, see, e.g., Figure 6) showed a Pearson correlation coefficient of 0.9817 (see Figure 7).

### 3.4. Yaw slope inter- and intra-individual variability

**Figure 8:**
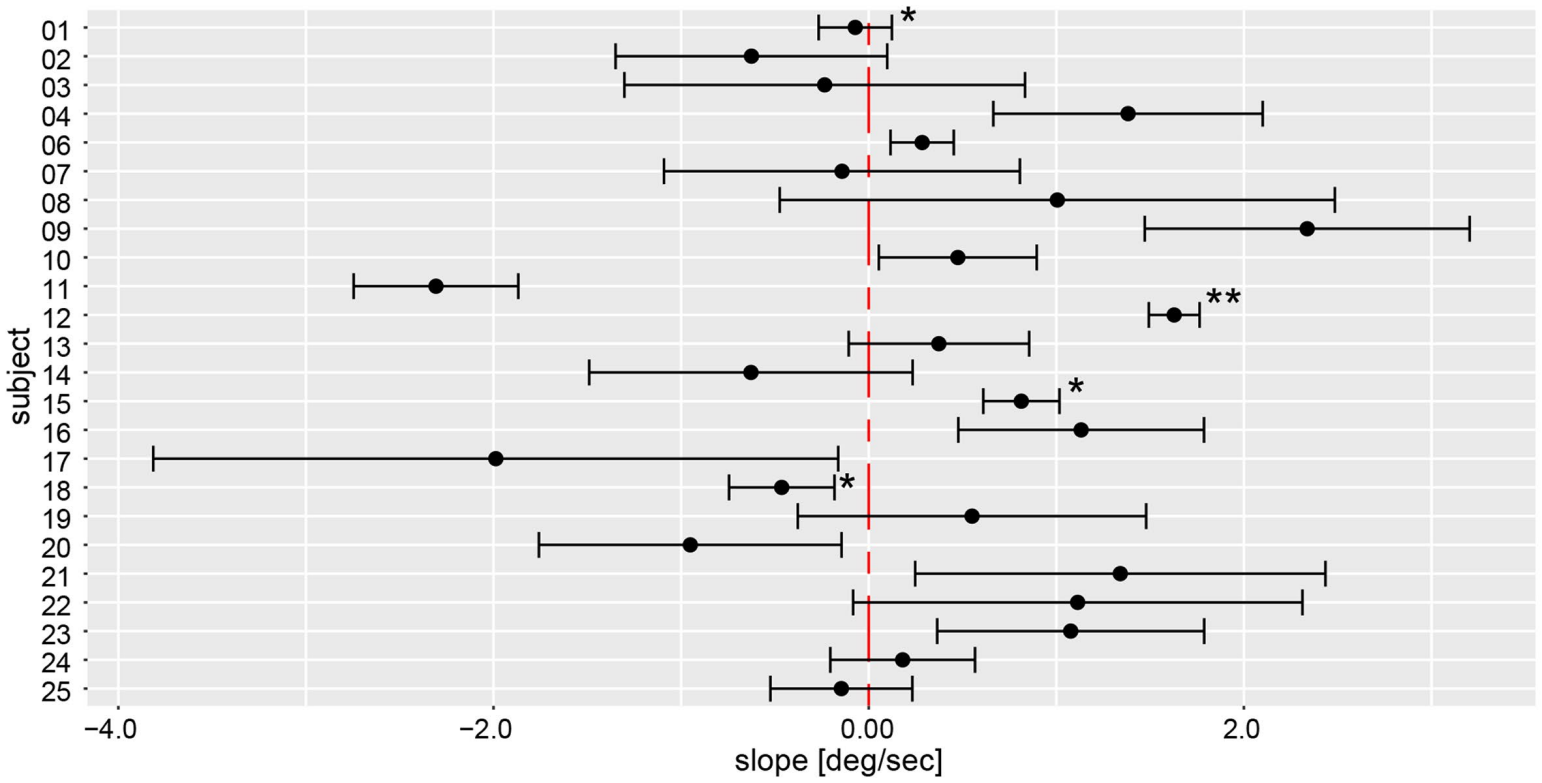
Illustration of individual average deviations (including 95% confidence levels) for every participant’s yaw slope for a total of 6 trials. The average values and confidence levels were calculated after outliers (i.e., trials exceeding a three times interquartile range) were removed. Individual average traces were marked with a star (“*”) to indicate that an outlier was removed, the number of stars reflects the number of outliers removed. The distribution of a participant is not significantly different to zero when the whisker crosses with the red, dashed zero-crossing line. Negative slope corresponds to rotation to the right, positive to the left.

Yaw slope as an indicator for deviation of chest heading demonstrated average (±1SD) slopes of 0.23 deg/s ± 1.31 deg/s (range of slopes −4.58 deg/s to 7.94 deg/s), with absolute slope values of 1.00 deg/s ± 0.88 deg/s. When assessing direction and magnitude of deviations, four participants deviated significantly to the right, nine participants to the left, whereas for 11 participants the distribution of yaw slope did not significantly differ from zero degrees (Figure 8). Different patterns in the two measurement approaches (manual angular detection vs. IMU angular rotation) can be seen in subject #6, shifting from a small but significant deviation to the right side (manual angular detection) to a significant deviation to the left side (IMU). The deviation of subject #25 becomes non-significant when considering the yaw slope instead of the manual measurements. Subjects #3, 7, 8, 9, 14, 17, 19, 21, and 22 showed a relatively large spread in yaw slope distribution, while subjects #1, 4, 12, 15, 18, 24, and 25 presented with a narrow distribution. Similar to the manual angle measurements, subject #17 yielded the highest variability between all trials.

On a trial-by-trial basis, we found that 7/24 subjects deviated to the same side on all 6 trials, whereas all other subjects deviated to the other side on 1 (*n* = 10), 2 (*n* = 5) or 3 (*n* = 2) trials. Intra-individual trial-to-trial variability (outliers removed) varied considerably between participating subjects (range = 0.13 deg/s to 1.74 deg/s), with an average value of 0.67 deg/s ± 0.41 deg/s. The intra-class correlation coefficient for the six repetitions (no outliers removed) demonstrated a moderate reliability of 0.61 (95% CI = 0.53–0.69, *p* < 0.001).

### 3.5. Movement dynamics – Inter-individual variability of relevant yaw deviation

**Figure 9:**
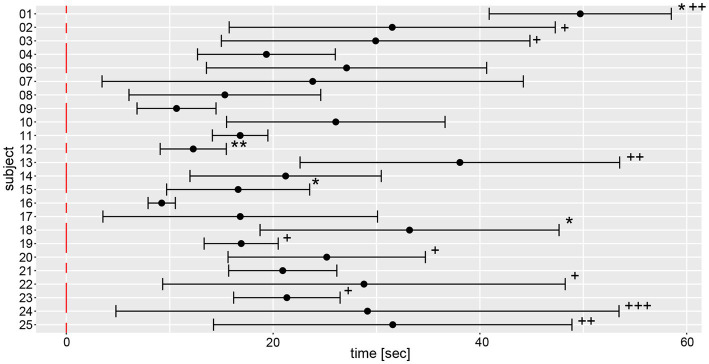
Illustration of individual average time to relevant yaw deviation (over a total of 6 trials) including 95% confidence levels. The average values and 95% confidence levels were calculated after outliers exceeding a three times interquartile range for yaw slope distributions were removed (Figure 7). Individual average traces were marked with a star (“*”) to indicate that an outlier was removed. Measurements without relevant yaw deviation within the recording period of 1 min were excluded and individual average traces were marked with a plus (“+”) for each such trial.

Analysis of movement dynamics showed an average time (±1 SD) to relevant yaw deviation of 22.1 s ± 12.3 s (range of onset time: 5.6–59.2 s) after onset of marching. In 15 out of 139 measurements, there was no relevant deviation of yaw after the total measurement duration of 60s (as indicated by a star (*) in Figure 9).

Average and width of the confidence levels of the time to relevant yaw deviation varied substantially between subjects, as reflected most obviously by subject #16 (presenting with the lowest mean and the smallest range) and subject #24 (having the highest range of time to relevant yaw deviation).

### 3.6. Movement dynamics – Yaw deviation behavior

**Figure 10:**
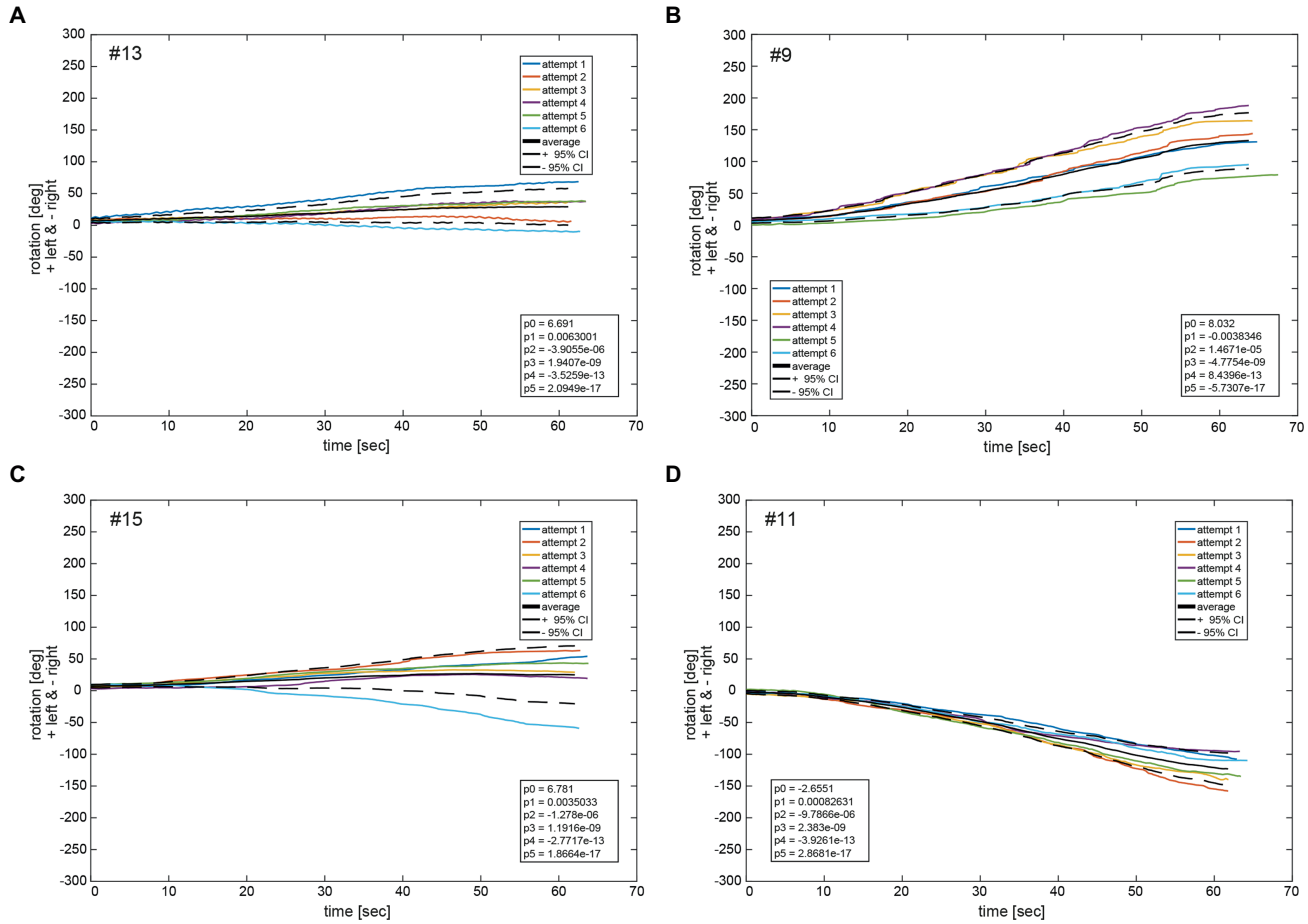
Illustrative examples of polynomial functions *p_x_* fitted on median filtered yaw per participant for the same four participants as shown in Figures 4, 5 (#13, #09, #15, and #11) in panels **A–D**. The black solid line depicts the average function f_av_ of all fit functions. The dashed black lines indicate the 95% confidence intervals for the total of 6 fit functions. The legend gives the coefficients of a 5th order polynomial *p_av_* of Equation (S1) $P_{5}\left(x\right)=p_{5}x^{5}+p_{4}x^{4}+p_{3}x^{3}+p_{2}x^{2}+p_{1}x+p_{0}$ fitted to the average function f_av_ of all fit functions *p_x_*. A negative angle corresponds to a rotation to the right, a positive to the left.

**Figure 11:**
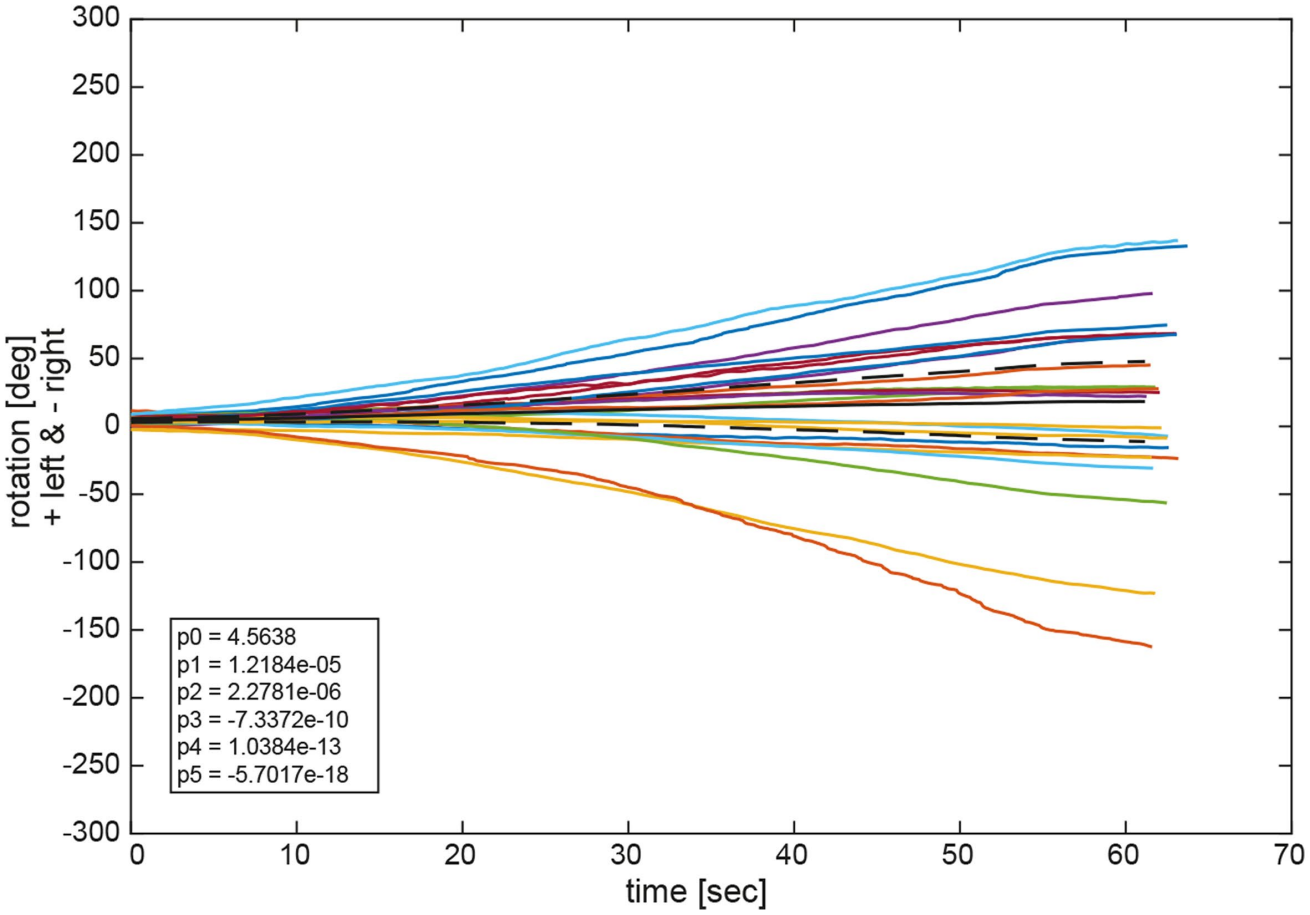
Average functions f_av_ of all polynomial functions *p_x_* for all trials per participant and for all participants. The black solid line depicts the average of all average functions f_av_av_. The dashed black lines indicate the 95% confidence intervals for the 24 individual average functions f_av_. The legend gives the coefficients of a 5th order polynomial of Equation (1) $P_{5}\left(x\right)=p_{5}x^{5}+p_{4}x^{4}+p_{3}x^{3}+p_{2}x^{2}+p_{1}x+p_{0}$ fitted to the average of all average functions f_av_av_. A negative angle corresponds to a rotation to the right, a positive to the left.

Figure 9 depicts the fitted polynomials for the median filtered yaw for all attempts of the same subjects as in Figures 4, 5. While subjects #13 (Figure 10A) and #15 (Figure 10C) are examples for a slight deviation to the left, subjects #9 (Figure 10B) and #11 (Figure 10D) represent the typical course for larger deviations to the left and to the right, respectively. Variability is always higher toward the end of the measurement than at the beginning as indicated by the increase of the confidence interval over time. Fitted polynomials for all 24 subjects can be found in the Supplementary material. The average alteration for all subjects over time indicates a tendency to left deviations, whereas 14 curves lie outside the 95% confidence interval (Figure 11).

Observation of the coefficients of the fitted polynomials depict a merely linear behavior of yaw deviation represented by the linear polynomial coefficient p1 being the largest of all polynomial coefficients, apart from p0 representing the y-intercept. Observation of average functions f_av_ for all participants shows a slight tendency toward the left side in deviation. This can also be observed in the average of all average functions f_av_av_ which slightly deviated in positive direction (left) in Figure 10. Specifically, coefficients of the 5th order polynomial function fit to f_av_av_ depicts a merely linear behavior represented in the largest coefficient of first order p_1_ with a slight tendency to the left represented by a small but positive value.

## 4. Discussion

Two different measurement setups were used to quantify angular orientation and to identify angular deviations relative to the subjective straight-ahead (SSA) in the Fukuda Stepping test (FST). Specifically, we assessed both static (i.e., angular deviation) and dynamic (i.e., slope of yaw rotation) aspects of a repetitive FST over 60 s combining traditional angular measurements and a chest-fixed inertial measurement unit (IMU). We noted excellent correlation between manual measurements and IMU-sensor based measurements, indicating that an IMU-sensory based approach may provide a valuable alternative to assess both static and dynamic aspects of the SSA while walking on the spot. An overall slight leftward bias was noted when calculating a signed average (4.1 deg ± 75.7 deg) with more subjects demonstrating significant angular rotations to the left (*n* = 8 (manual measurements) and *n* = 9 (IMU-based measurements), respectively) than to the right (*n* = 7 (manual measurements) and *n* = 4 (IMU-based measurements)), pointing to a potential overall leftward directional bias in the SSA. However, a significant fraction of participants did not demonstrate deviations to either side (*n* = 9 (manual measurements) and *n* = 11 (IMU-based measurements)), underlining the individual differences in task performance. Furthermore, also within subjects the reproducibility of the FST was moderate at best with an average trial-to-trial variability of 38.9 deg ± 24.1 deg (range = 3.0–103.4 deg) and an intraclass correlation coefficient of 0.61 (95% CI = 0.53–0.69). In those participants demonstrating significant deviations over time, the yaw slope followed a linear behavior.

### 4.1. The dynamics of angular deviation while performing the FST using an IMU-based approach and comparison with manual measurements

Here we provide for the first time a quantitative analysis of the dynamics of angular deviation during the FST by use of a single IMU sensor attached to the participant’s trunk and a comparison of its performance to the traditional approach of manually measuring the angular deviation at the end of the trial. Overall, both recording setups (IMU-sensor based assessment vs. manual measurements) were able to catch angular deviations reliably with an excellent trial-by-trial correlation (Pearson correlation coefficient = 0.98) between the two approaches. Thus, using an IMU-sensor based setup for recording may provide a valuable alternative to the traditional angular deviation measurement at the end of trials. While our approach provides a yaw-slope as indicator of trunk rotation (reflecting angular velocity), it does not allow the calculation of the total angular deviation at the end of the trial due to growing offsets over time of the sensory input. This limitation can be potentially resolved by fusing the magnetometer data (not considered for the current analysis) and the gyroscope data, which should be addressed in future studies in the field. However, the dynamics of angular deviation can be studied with high temporal resolution, demonstrating additional valuable parameters as time of onset of angular deviation and the behavior of deviation (linear vs. non-linear). Thereby different phases of body yaw orientation relative to the SSA can be observed. Specifically, a first phase of fairly stable angular alignment with the SSA is followed by a second phase with a mostly linearly growing angular deviation (but with considerable in between subject and within-in subject variability). Thus, the angular deviation at the end of the trial will be affected both by the time to angular rotation onset and the steepness of the slope. Therefore, it is of little or no surprise that paradigms with shorter trial duration and/or fewer steps demonstrate smaller angular deviations. For future studies (also in patients), such an IMU-sensory based approach allows retrieval of a more detailed pattern in individual biases in straight-ahead. This may further help to delineate differences in SSA in healthy human subjects and patients with lesions along the graviceptive pathways. Specifically, parameters such as time to angular rotation onset, the steepness of the slope and the variability over a series of trials may provide useful additional information to distinguish patients from healthy controls. Qualitatively, the direction of drift was identical in all but one subject (subject #6) when comparing both measurement techniques. We can only speculate about the reason for these differences. Noteworthy, absolute drifts in subject #6 were small, thus making it more prone to noise in the IMU measurements or also errors in manual measurements of angular deviation.

Previously a setup for quantitatively measuring the subject’s performance during the FST was proposed by Belluscio and colleagues (24), retrieving 3D linear accelerations and angular velocities from a total of five IMU sensors located at the occipital cranial bone, the sternum, at L4/L5 level, and at the lateral malleoli (left and right foot). While these authors report that with the instrumented FST (iFST) they could reliably distinguish motor control patterns in patients and controls, they did not use IMU sensor data to calculate the angular deviations resulting from the FST. Thus, a direct comparison with the sensor-based approach proposed here in healthy human subjects is not possible.

### 4.2. Comparison of the accuracy and directional preponderance of the FST in our study with findings from the literature

In our study subjects performed the FST over a period of 60 s at their own pace, resulting in overall (average ± 1SD) absolute deviations of 58.0 deg ± 48.6 deg. When taking the direction of rotation into account, individual signed deviations averaged at 4.1 deg ± 75.7 deg. Fukuda originally proposed that after 100 steps the angle of rotation does not exceed 45 deg (7). Compared to the literature (manually measured angular deviations at the end of trials, no IMU-sensor based measurements), reporting absolute angular deviations ranging from (16.1 ± 12) deg [60 s of stepping (25)] and 16 deg ± 19 deg [for 30 s stepping (9)] to 30 deg ± 22 deg [50 steps, (14)] and 45 deg [for 60 s of stepping; median value (15)], larger average angular deviations were noted in our study. This was true even for the same number of steps. However, considerable variability was noted between studies. This was also true for signed average deviations (with a positive sign indicating leftward deviations and a negative sign indicating rightward deviations), which ranged between 0.3 deg ± 20.4 deg [for 60 s stepping (25)], 17.8 deg ± 105 deg [for 100 steps, (19)] and 24 deg ± 71 deg [for 100 steps (18)] in single studies, demonstrating similar values than in our study. In another study that reported on subgroups with leftward and rightward deviations separately, very similar deviation angles were noted [for 45 steps; 29 deg ± 10 deg vs. −30 deg ± 9 deg (26)]. Comparisons, however, need to be made with caution as there are variations in the way the FST was performed, i.e., requiring either a certain trial duration (either 30 or 60 s) or a certain number of steps to be performed (ranging from 30 to 100 steps). Furthermore, in most studies, left-handed subjects were pooled with right-handed subjects.

Using different approaches to define significant angular deviations while performing the FST, we observed varying rates in our subject population that met these criteria. Specifically, subjects deviated more than 10 deg from their starting position in 90.6% of trials, resulting in 21/24 subjects (88%) demonstrating average deviations of more than 10 deg. Using a more conservative approach and requiring that the 95% CI of the individual average value does not include zero, we noted significant deviations in 15/24 participants (63%) when performing manual measurements at the end of the trials. When relying on the IMU sensor data and fitted yaw slopes, the fraction of patients with significant deviations (again requiring that the 95% CI of the individual average do not cross zero) was 13/24 (54%). In the published literature, a majority of participants demonstrated significant angular rotations (with varying definitions of what was considered “significant deviations”) during the task, with reported fractions of 62% (9), 93% (27), 96% (28), 97% (26), and 98% (15). Thus, the fraction of patients considered to shift significantly to either side strongly depends on the analysis performed and how a significant shift is defined.

The individual distribution of significant directional changes (leftward vs. rightward; 33% vs. 29% in our study) seems to be highly variable in the literature, with some studies reporting larger fractions of participants with leftward deviations than rightward deviations [34% vs. 28% (9); 59% vs. 37% (28); 60 vs. 40% (25)], whereas in others leftward deviations were less frequently observed than rightward deviations [43% vs. 54% (26) or 30% vs. 63% (27)]. Yet other studies reported fractions of subjects rotating rightward and leftward, respectively, that were almost of equal size (50.4% vs. 47.7%) (15). Taking into account handedness, Peitersen emphasized the preponderance of right-handed subjects to rotate to the right (29). This was later confirmed by Reiss and Reiss, reporting that 70% of right-handed participants deviated toward the right side, whereas only 28% deviated to the left (18). For left-handed subjects, such directional preponderances were less pronounced, but still showing a larger fraction of participants deviating rightward (54%) than leftward (42%) (18).

Variability noted in between studies could emerge from various causes, including differences in instructions (e.g., to which extent participants were told to lift their knees and by which pace they should be stepping), potential external auditory cues that may serve as a reference and thus bias turning direction, and fatigue on repeated testing. While our participants did not receive any feedback about their trial performance, this may have also biased task performance in previous studies.

Taken together, there is significant heterogeneity among studies regarding the direction and magnitude of angular deviation in the FST in healthy human subjects without an obvious preference in direction. Whether handedness affects directional preponderance remains debatable with a single study comparing fractions among left-and right-handed participants only (18). While we see an overall (albeit minor) leftward deviation during repeated FST in our study, which is consistent with the leftward deviations noted when walking straight-ahead blindfolded (baseline trials), as reported previously by our group (16), it remains open whether it is a random effect or not. Furthermore, and as emphasized before by other studies, the FST seems to be insensitive to detect peripheral-vestibular deficits. For example, Zhang and colleagues reported that patients with acute unilateral vestibular deficits showed similar fractions of deviation (ipsilesional vs. contralesional) (30).

### 4.3. Trial-to-trial variability (i.e., the precision) of the FST – Comparison with the literature

Lateralized hemispheric functions (represented by handedness and footedness) have been linked to preferential deviations toward one side in some studies (18, 27, 29), while others found more balanced distributions (15). This finding raises the question how consistent trial performance in single subjects over time is, thus how much intra-individual variability there is.

Over the six repetitions of the FST (and after removing outliers), individual subjects in our study demonstrated considerable variability in their task performance, both with regards to the direction of deviation and the magnitude of angular deviation. Specifically, only 11/24 participants deviated to the same direction (leftward vs. rightward, manual measurements) over all repetitions, whereas the remaining 13 subjects demonstrated deviations to either the left or the right side, albeit with a preference to one side. Thus, the resulting trial-to-trial variability, reflecting the precision in the FST, varied substantially among subjects as well. While some participants were able to repeat the FST with very high precision (reflected by a trial-to-trial variability of as little as 3.0 deg), other subjects demonstrated much more variability in their task performance, resulting in a trial-to-trial variability of up to 103.4 deg. This large inter-individual difference in precision of the FST is reflected in the overall average SD between trials of 38.9 deg ± 24.1 deg.

The reasons for the observed substantial inter-individual variation in task performance remain to be discussed. While the task performance in individual participants may have been negatively affected by random noise and fatigue, the high precision achieved by some subjects speaks against a given limitation by the sensory systems involved. Thus, it is indeed possible to repeat the FST with high precision. However, it remains open why we noted so much variability among our participants. With regards to the proposed random noise, a predominance of very low frequencies is predicted, as otherwise a different drift pattern, looking more like a random walk and less like a gradual drift would be expected [as previously described by others for trial-to-trial variations in the yaw VOR (31)].

Overall, in our study we confirmed previous observations that both the accuracy and the reproducibility of the FST over time in healthy human subjects vary substantially. With regards to potential underlying mechanisms to explain the task performance observed in a group of subjects, two hypotheses have been introduced in this article. While individual differences in the orientation and the configuration of the macular organs and in central processing of vestibular input would predict that such asymmetries are stable over time in individual subjects, asymmetries arising from random noise in the sensory or motor systems would demonstrate a low reproducibility over time. Noteworthy, previously a static bias in perceived rotation has been demonstrated in healthy human subjects. Specifically, when asked to null out pseudorandom rotational perturbations in darkness in order to remain perceptually stationary, most participants showed a slow linear drift of velocity to one side (32). The authors concluded that their observation is consistent with a small, constant imbalance of vestibular function, being of either peripheral or central origin.

In the light of previous observations (32) and the individual patterns observed in our study sample with a moderate intra-class correlation coefficient of 0.61, neither of the two hypotheses can fully explain all the participants’ behavior in our study. While some participants demonstrated no significant deviations from the SSA but had large trial-to-trial variability, others presented with large deviations but high reproducibility of the FST. Yet others were both precise and accurate in the FST or were both inaccurate and imprecise. Thus, likely on top of individual differences in the orientation and configuration of the macular organs and in central processing of vestibular input, random noise in the sensory or motor input integrated or other, not identified mechanisms contributed to the highly variable pattern observed. Other subject-specific factors that could lead to such subject-dependent stepping in place asymmetries include anatomical variations in lower limb configuration Specifically, differences in leg length, variations of the degree of pitch or roll of pelvis orientation, the angle between the thigh and the lower leg could lead to slight difference in the placement of the feet with each step. Noteworthy, we have not assessed or controlled for such anatomical variations without any disability in daily life. Thus, this should be further assessed in future studies in the field.

When comparing our results with those provided in the literature, again significant variability can be observed. Such variability, however can be explained at least partially by differences in the paradigm (i.e., varying number of steps per trial). Noteworthy, the study with the most similar paradigm (28, 33) than the one used here (we estimate about 60–80 steps over the 60s stepping period as required in our study), demonstrated an average trial-to-trial variability (*n* = 3 trials, 100 steps each) in a comparable range, averaging at 45 deg ± 36 deg (range 2–150 deg) (28, 33). In another study with 3 repetitions and only 50 steps per repetition, average trial-to-trial variability was substantially lower, averaging at 24.3 deg ± 14.5 deg (14). With only two repetitions (being 4 h apart) and requiring only 30 steps per repetition, Jordan reported a trial-to-trial variability of 40.8 deg ± 39.7 deg (34), being higher than in most other studies.

With regards to the direction of rotation, again in the literature varying performance of healthy participants have been reported. Whereas according to Peitersen “nearly all rotated into changing directions” on repeated testing after 2 h (29), Reiss and Reiss found consistent deviations to the same side in 80/86 subjects on repeated testing 3 months apart, however, did not report on the trial-to-trial variability (18). Previc and colleagues (collecting two repetitions of the FST in the same session) reported a modest intraclass correlation coefficient of 0.54 (15), largely exceeding the value of −0.04 reported by Jordan (29), but slightly smaller than the value of 0.61 (range from 0.53 to 0.69; reflecting moderate reliability) obtained in our study for either measurement approach. Test-re-test reliability on two consecutive days (24 h apart) was studied by Bonanni and colleagues (19), reporting an intra-class correlation coefficient of 0.52 for the 100 step paradigm and of 0.66 for the 50 step paradigm. The reason for these intra-and inter-individual differences and sometimes substantial deviations when performing the FST have not been fully elaborated. They may be linked either to the vestibular organs, to central aspects such as hemispherical lateralization in the processing of vestibular input or random noise.

### 4.4. Limitations of this study

There are several study-specific limitations that need to be discussed here. On one hand, fatigue may have played a significant role, as data collection for the FST (six trials in sequence with breaks in between) were obtained in the same session (but always after) as a repetitive walking task, previously reported in detail (16). Looking at the distribution of outliers (*n* = 5 trials) in our data set, however, there was no accumulation of outliers toward the end of the session, suggesting that fatigue may have rather played a minor role only if at all. Furthermore, while we limited FST trial duration to 60s, we did not control for the stepping pace, resulting in a varying number of steps performed from trial to trial. Thus, individually different numbers of steps may have contributed to the considerable inter-individual and intra-individual variability observed in our study, as previously it has been shown that with increasing stepping size variability and deviations increased (19).

## 5. Conclusion

The ability to keep the body aligned with the straight-ahead direction during the FST (i.e., walking on the spot for 60 s with visual and auditory cues removed) varied substantially in healthy human subjects, with a majority of subjects demonstrating significant shifts in the subjective straight ahead that often exceeded the limits originally proposed by Fukuda (7). Most participants consistently deviated to either the left or right side on repetitive testing, suggesting the presence of an individually varying directional bias in SSA. However, with a moderate intraclass correlation coefficient only and a trial-to-trial variability that reached almost 40 deg on average, we noted substantial intra-individual variability, which likely emerged from random noise. Thus, our findings suggest that likely both individual differences in the orientation and the configuration of the macular organs and in central processing of vestibular input and that random noise contributes to the pattern of individual angular deviations in the FST observed here. In addition, other, yet unidentified extra-vestibular anatomical factors including lower limb configuration may contribute as well to the asymmetries observed. Furthermore, using a trunk-based IMU-sensor allowed capturing angular deviations reliably (significantly correlating with classic angular measurements after the FST) and at the same time also provided an assessment of the dynamics of angular orientation including time of onset of deviation and the deviation pattern. Thus, we found a mostly linear pattern of angular deviation over time in heathy subjects. In future studies, these new dynamic parameters should be investigated in different patient groups as well to assess their value in the diagnostic workup.

## Data availability statement

The raw data supporting the conclusions of this article will be made available by the authors, without undue reservation.

## Ethics statement

The studies involving human participants were reviewed and approved by Ethikkommission Nordwest- und Zentralschweiz (EKNZ, ID = 2020-01712). The patients/participants provided their written informed consent to participate in this study.

## Author contributions

SH conception of the work, data analysis, interpretation of data for the work, and revising the work critically for important intellectual content. VDdC acquisition, analysis and interpretation of data for the work, drafting the work and revising it critically for important intellectual content. DB main responsible for data analysis, interpretation of data for the work, and revising the work critically for important intellectual content. AT conception of the work, data analysis interpretation of data for the work, and drafting the work and revising it critically for important intellectual content. All authors approved the final version of the manuscript and agreed to be accountable for all aspects of the work in ensuring that questions related to the accuracy or integrity of any part of the work are appropriately investigated and resolved. The authors confirm that all persons designated as authors qualify for authorship, and all those who qualify for authorship are listed.

## Funding

VDdC was financially supported by the Cantonal Hospital of Baden (grant #009192).

## Conflict of interest

The authors declare that the research was conducted in the absence of any commercial or financial relationships that could be construed as a potential conflict of interest.

## Publisher’s note

All claims expressed in this article are solely those of the authors and do not necessarily represent those of their affiliated organizations, or those of the publisher, the editors and the reviewers. Any product that may be evaluated in this article, or claim that may be made by its manufacturer, is not guaranteed or endorsed by the publisher.
