# Supplementary material for: Test-re-test reliability and dynamics of the Fukuda–Unterberger stepping test
Source: Front Neurol. 2023 Mar 29;14:1128760. doi: 10.3389/fneur.2023.1128760 (PMC10090507; doi:10.3389/fneur.2023.1128760)

# Supplementary material

## Polynomial fitting of individual trials

For the analysis of the yaw deviation dynamic behavior per participant the average function f_av_ and the 95% confidence intervals of all polynomial functions *p_x_* fitted to the median filtered yaw for a total of 6 measurements were computed in Matlab using equation (S2). A polynomial of 5^th^ order of equation (S1) *p_av_* was fitted to the computed average function f_av_.

(S1) $P_{5}\left( x \right)=p_{5}x^{5}+p_{4}x^{4}{+p}_{3}x^{3}+p_{2}x^{2}{+p}_{1}x+p_{0}$

(S2)

% p: matrix of 2 columns; rows: discrete values for fitted polynomial functions p_x_ for all measurements per participant

for k = 1: lengthShortestFittedPolynomialFunction

% average of fitted polynomials

f_av(k)= mean(p(k,:));

% confidence interval 95%

N = length(p(k,:));

ySEM = std(p(k,:))/sqrt(N); % standard error of the mean of all fitted functions at each value of x

CI95 = tinv([0.025 0.975], N-1); % 95% probability intervals of t-distribution

yCI95 = bsxfun(@times, ySEM, CI95(:)); % 95% confidence intervals of all fitted functions at each value of x

end

Finally, for analysis of yaw deviation dynamic behaviour over all participants the average function f_av_av_ and 95% confidence intervals of all average functions f_av_ per participant was computed in Matlab similar to (S2). A polynomial of 5^th^ order of equation (S1) *p_av_av_* was fit to the computed average function f_av_av_.

## Correlation analysis of angular deviation: manually measured angles vs. calculated yaw slopes

The yaw slope as a measure to describe upper body rotations was compared to the manually measured rotational deviations in a correlation analysis. The 95% confidence levels of manually measured heading rotation angles $angle\_manual$, yaw slope $slope$ and time to relevant yaw deviation $t\_rel\_change$ distribution data for all measurements of every subject were calculated in R as shown in (S3). For all confidence level computations, the average values and confidence levels were calculated without outliers exceeding a three times interquartile range for the variable “yaw slope”.

(S3) $confInt<-marching.data \%>\% group\text{\_}by\left( participant \right) \%>\%$

$$n=n\left( measurements \right),ave=mean\left( angle\_manual \left| slope \right| t\_rel\_change \right), std=sd\left( angle\_manual \left| slope \right| t\_rel\_change \right),$$

$$error=qt\left( 0.975,n-1 \right)*{std}/{sqrt\left( n \right)}$$

$$left=ave-error,right=ave+error$$

Resulting fits are shown in Figure 7 (main manuscript), demonstrating a significant correlation with a Pearson correlation coefficient of 0.9817.

## Fitted polynomials per participant

Polynomial functions *p_x_* fitted on median filtered yaw per participant for all 24 participants (#1-4; #6-25). The black solid line depicts the average function f_av_ of all fit functions. The dashed black lines indicate the 95% confidence intervals for the total of 6 fit functions. The legend gives the coefficients of a 5^th^ order polynomial *p_av_* of equation (S1) $P_{5}\left( x \right)=p_{5}x^{5}+p_{4}x^{4}{+p}_{3}x^{3}+p_{2}x^{2}{+p}_{1}x+p_{0}$ fitted to the average function f_av_ of all fit functions *p_x_*. A negative angle corresponds to a rotation to the right, a positive to the left.


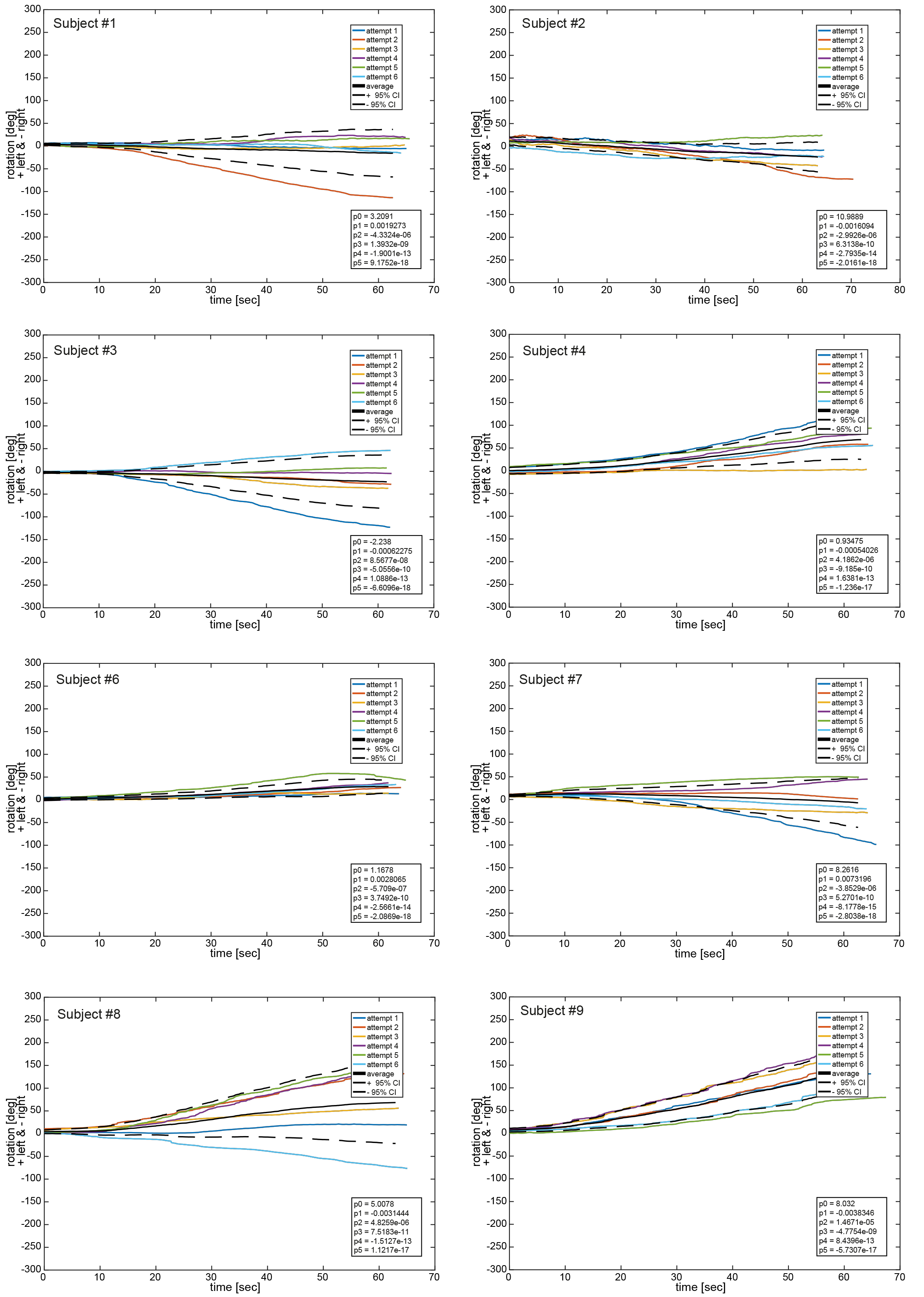


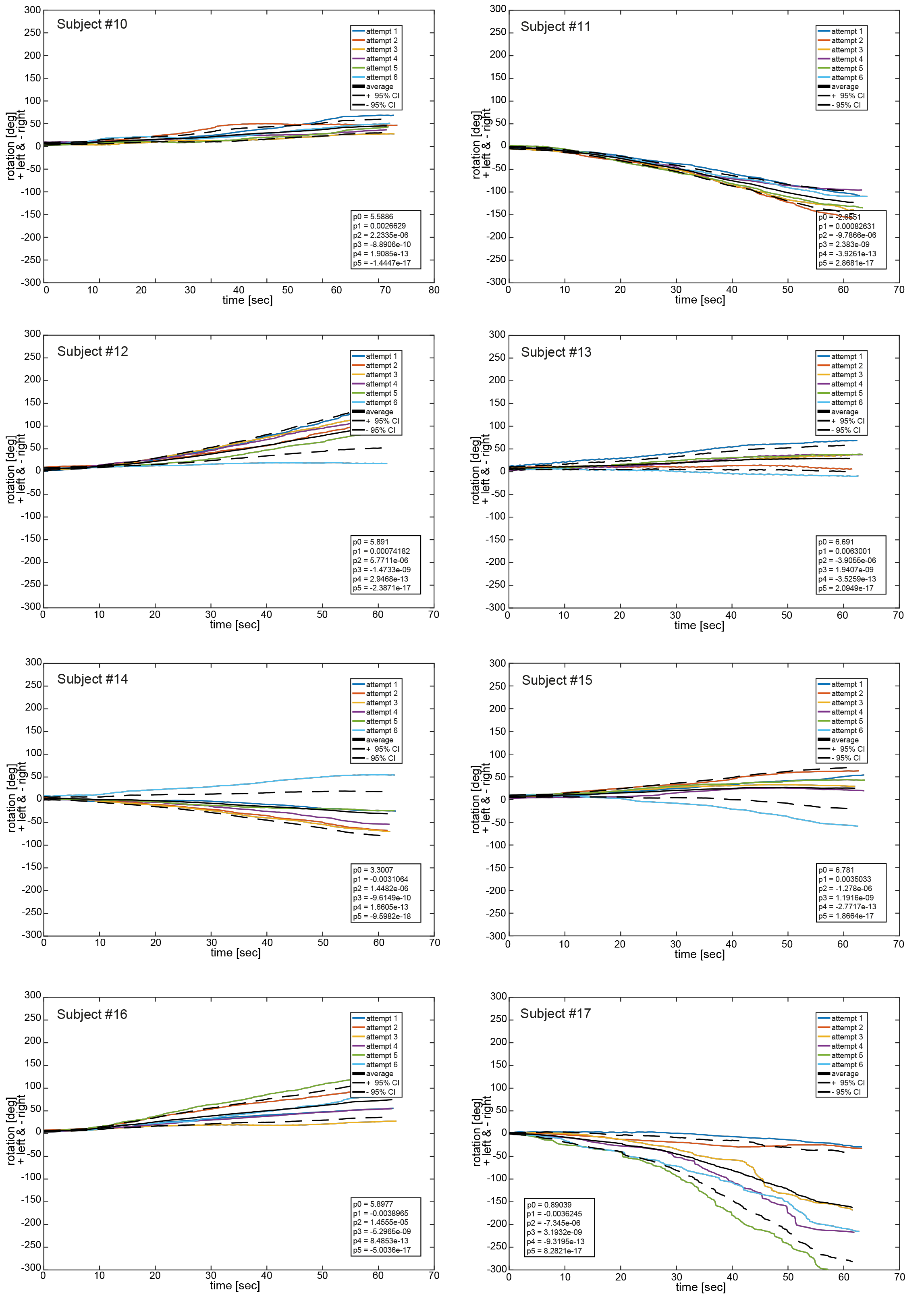


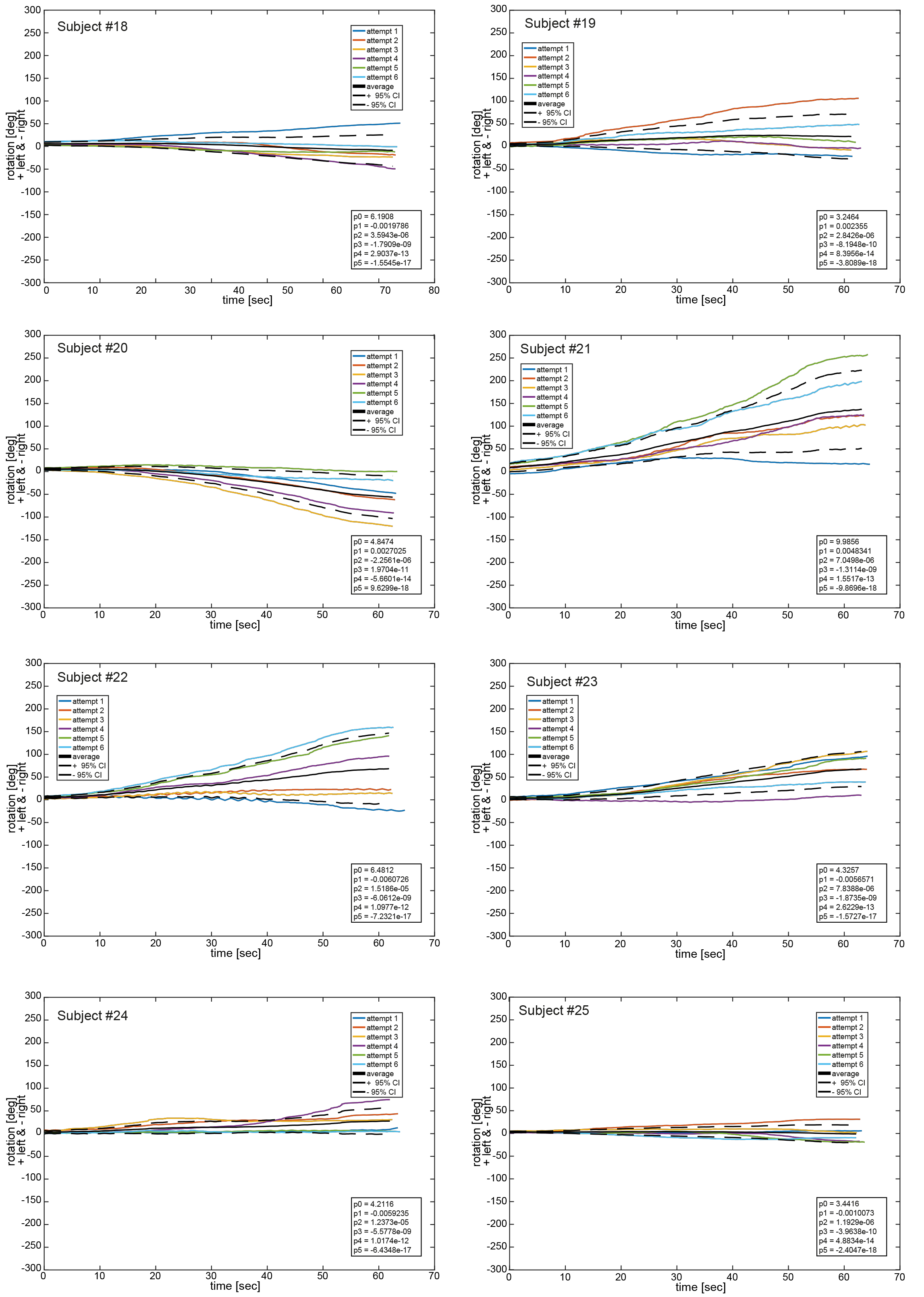

Supplement: Supplementary file 1 [file Table_1.DOCX]
